# Acceptance, Adherence, and Side Effects of Depot Medroxyprogesterone Acetate: A Prospective Observational Study

**DOI:** 10.7759/cureus.58700

**Published:** 2024-04-21

**Authors:** Rajalakshmi Rajaraman, Sasi Vaithilingan, Thirumalaichiry S Selvavinayagam

**Affiliations:** 1 Community Health Nursing, Vinayaka Mission's Research Foundation (DU), Salem, IND; 2 Child Health Nursing, Vinayaka Mission's College of Nursing, Puducherry, IND; 3 Child Health Nursing, Vinayaka Mission's Research Foundation (DU), Salem, IND; 4 Epidemiology and Public Health, Directorate of Public Health and Preventive Medicine, Chennai, IND

**Keywords:** adherence to contraception, injectable contraceptive, hormonal contraception, depot medroxyprogesterone acetate, contraceptive acceptance, contraceptive side effects, long-acting reversible contraception

## Abstract

Introduction

In India, one of the world’s most populous and swiftly growing countries, it is crucial to prioritize the utilization of safe and effective contraception, as contraceptive strategies play a pivotal role in bolstering community health. It is widely acknowledged that ensuring appropriate timing and spacing of pregnancies is crucial for the well-being of reproductive, maternal, neonatal, child, and adolescent health. Adoption of reversible or spacing contraceptive methods can significantly enhance women’s health outcomes by reducing the occurrence of undesired, closely timed, and mistimed pregnancies. Consequently, in response to the pressing need for dependable contraception in India, this study seeks to assess the acceptance, adherence, and side effects of the injectable contraceptive depot medroxyprogesterone acetate (DMPA) among its users.

Methods

This prospective observational study was done at the State Government Taluk Hospital in the Cuddalore District of Tamil Nadu from July 2022 to October 2022. A total of 40 women of reproductive age who opted for DMPA as their contraceptive method and met the inclusion criteria were recruited through a purposive sampling method. A structured questionnaire was used to collect the data.

Results

The majority of the participants were women aged 21-25 years (n=14; 35%). The participants were predominantly Hindu (n=39; 97.5%), and 35 (87.5%) had completed higher secondary education. All participants (n=40; 100%) resided in rural areas and the majority were homemakers. A significant proportion of the participants had two children (n=21; 52.5%), and all of them received information on DMPA primarily from health personnel. At the initial point of data collection, three-fourths of them took the first dose (n=13; 32.5%) and only a few took more than three doses (n=3; 7.5%). In the third month, the results showed a drop in DMPA use, which indicates a lower adherence particularly linked to side effects like irregular bleeding (n=15; 37.5%) and amenorrhea (n=9; 22.5%). Furthermore, 35 (87.5%) of the women chose DMPA for birth spacing due to its efficacy and convenience, with few initiating it during postpartum (n=4; 10%) and post-abortal (n=1; 2.5%) periods. The reasons for continuing DMPA use included efficacy (n=20; 50%), discreet usage (n=15; 37.5%), and curiosity (n=13; 32.5%). Half of the participants reported no side effects. The study identified associations between DMPA users and the number of living children and occupational status inferring that DMPA contraception is used for spacing births.

Conclusion

The results of this study imply that the use and adherence to injectable contraceptive DMPA need to be strengthened among rural women. Thus, the study suggests incorporating information, education, and communication strategies, to enhance awareness among rural women about injectable contraceptives.

## Introduction

The global challenge of an increasing world population, with an estimated 7.9 billion individuals and 26 children being born every second, as highlighted by the United Nations, is intricately linked to the population dynamics of India [1]. As the second-most-populated country, the population of India is rapidly increasing and is anticipated to reach 1.5 billion by the year 2050 [1-3]. This demographic surge underscores the critical need for responsible family planning, and potentially an inter-birth interval of at least three years. Addressing the inter-birth challenge has become crucial not only for India but also in the broader context of global population dynamics [4-6], within which the effectiveness of different contraceptive methods plays a pivotal role. This effectiveness is not solely dependent on capacity to prevent pregnancy but also on consistency and proper usage over time. Choosing the most appropriate birth control method is contingent upon individual needs and willingness, as well as recognizing the unique advantages and disadvantages associated with each method. This ongoing evolution in contraceptive practices reflects society’s commitment to advancing reproductive health and empowering individuals to make informed choices for family planning and aligns with the need for responsible population management in the face of the global challenge [7-10].

This global context sets the stage for understanding the regional variations in the prevalence of injectable contraception, which is currently at 3.5%, however, regional prevalence varies significantly, with figures such as 15% in Sri Lanka, 10% in Nepal, 7% in Bangladesh, 5.9% in Bhutan, and 2.7% in Pakistan. Notably, the utilization of depot medroxyprogesterone acetate (DMPA) in India remains remarkably low at 0.2%, as reported by National Family Health Survey-5 (NFHS-5) data from 2020-2021 [11-13]. Despite a handful of studies on DMPA, there is a lack of comprehensive research exploring the global prevalence of injectable contraceptives. Even with the introduction of DMPA in Tamil Nadu through the Antara scheme, which is provided free of cost in all government hospitals since 2017, there has been minimal research conducted regarding the acceptance and adherence toward DMPA in Tamil Nadu. This study is the pilot test aimed to assess the acceptance, adherence, and side effects of DMPA among DMPA users. Through this investigation, we seek to determine the gap surrounding injectable contraceptives within its user population [14-16].

## Materials and methods

The study was approved by the Institutional Ethics Committee of Vinayaka Mission’s College of Nursing, Puducherry, India, under approval number VMCN PDY/IEC 2022/072. Ethical clearance was obtained from the Joint Director of Health Services, Department of Health & Family Welfare, Cuddalore District. This prospective observational study focuses on reproductive-age women who had chosen DMPA as the method of birth control and the study was conducted at State Government Taluk Hospital in the Cuddalore District of Tamil Nadu, a secondary care facility providing comprehensive family welfare services. The sample size calculated for the main study was 371. For the pilot study, 1/10th of the main study population was considered with 10% attrition. Hence the computed sample size for the pilot study was 40. The purposive sampling technique was employed to select the participants who fulfilled the inclusion criteria. The study included women aged 21-45 years, opting for DMPA as birth control and seeking contraception during the postabortal or postpartum period for maintaining intervals between births. Additionally, both literate and illiterate women were included in the study. Conversely, women under the age of 21, those with acute illness/ medical or surgical conditions, and not willing to participate were not included in the study. Informed written consent was obtained from all study participants after a thorough explanation of the study’s objectives and procedures using a participant information sheet. The data from the study participants were collected from July 2022 to October 2022.

A self-administered structured questionnaire was employed to gather data. The questionnaire was developed based on the guidelines [15,17] and it has two parts. The first part focused on the demographic characteristics of DMPA users comprising 11 items: age, religion, residence, educational status, occupational status, number of living children in the family, the primary source of information on DMPA, a health professional who administered the injectable contraceptive, a health center in which DMPA was received, and information regarding dose and initiation of contraception. The second part of the structured questionnaire was focused on acceptance and adherence, and the side effects of DMPA. The third part of the questionnaire investigated the reasons behind both the continuation and discontinuation of DMPA usage. It includes a checklist comprising six items for continuation and 11 items for discontinuation. The reasons for discontinuation were categorized as physiological factors (seven items), social factors (two items), and economic factors (two items). The questionnaire underwent rigorous review, validation by experts, and reliability testing using the test-retest method, yielding an “r” value of 0.76 which shows that the study questionnaire was reliable to use.

By adopting the interview technique, the data were collected from the participants using the constructed questionnaire. At the initial point of data collection, the study participants who came for DMPA injection were observed for acceptance and adherence to DMPA. Acceptance was determined based on the adoption of the first dose and adherence was determined based on the subsequent doses. They were also asked about the reason for the continuation or discontinuation of DMPA and side effects of DMPA. Three months after the study, the participants were observed for adherence to DMPA and the reason for continuation or discontinuation of DMPA using the same questionnaire and side effects of DMPA.

Statistical analysis 

Data were entered using EpiInfo version 7.0 and IBM SPSS Statistics for Windows, Version 28 (Released 2021; IBM Corp., Armonk, New York, United States) was employed for data analysis. Categorical variables are presented using frequency and percentage. Statistical associations between acceptance, onset, and adherence of DMPA were assessed using the chi-squared test, with p<0.05 indicating statistical significance.

## Results

During the period of data collection, 341 reproductive women attended the family planning clinic out of which 196 women reported current use of contraceptives. Of that, 32 (16.3%) chose an intrauterine device, 25 (12.8%) underwent tubectomy, 44 (22.4%) used condoms, 46 (23.5%) opted for DMPA, 36 (18.3%) opted for Chhaya, 8 (4.1%) were treated with emergency contraceptive, and only 5 (2.6%) used oral pills. From the above findings, it was observed that the acceptability rate for DMPA was 23.5%. Out of 46 DMPA users, 40 of them who fulfilled the inclusion criteria were involved in the study.

Among 40 DMPA users, a significant proportion of participants (n=14; 35%) fell within the age range of 21-25 years. The majority of participants were Hindu (n=39; 97.5%), 35 (87.5%) had completed higher secondary education, and all participants (n=40; 100%) resided in rural regions and the majority were homemakers (n=34; 85%). Among the respondents, 21 (52.5%) had two children, and all participants reported health personnel as their primary source of information on DMPA (Table 1). 

**Table 1:** Demographic characteristics of DMPA users DMPA: Depot medroxyprogesterone acetate

Variable n=40	Category	Frequency	%
Age (years)	21–25	14	35.0%
	26–30	10	25.0%
	31–35	10	25.0%
	36–40	6	15.0%
Religion	Hindu	39	97.5%
	Muslim	1	2.5%
Residence	Rural	40	100.0%
	Urban	0	0
Educational status	High school	35	87.5%
	Middle school	5	12.5%
Occupational status	Shop & market sales workers	1	2.5%
	Agricultural	5	12.5%
	Homemaker	34	85%
Number of living children in the family	1	9	22.5%
	2	21	52.5%
	≥3	10	25.0%
Primary source of information on DMPA	Health personnel	40	100.0%
Health professional who administered the injectable contraceptive	Medical officer	40	100.0%
Location where DMPA was received	District hospital	40	100.0%

**Figure 1:**
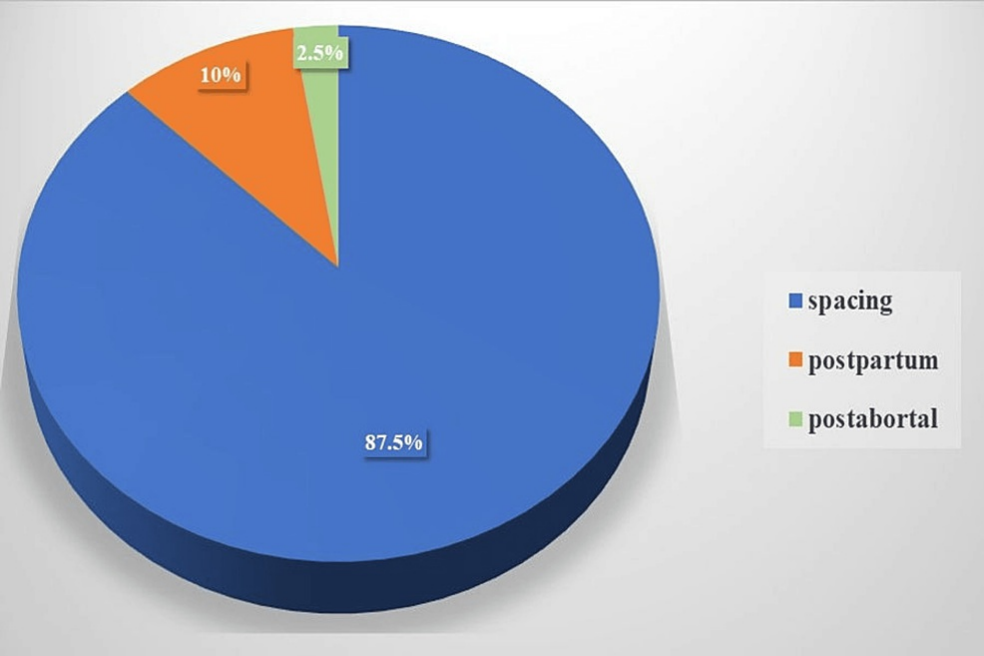
Reasons for DMPA initiation DMPA: Depot medroxyprogesterone acetate

Among 40 DMPA users, the majority (n=35; 87.5%) chose injectable contraception (DMPA) for the birth spacing interval due to its efficacy and convenience. Only a few initiated DMPA during the postpartum (n=4; 10%) and post-abortal (n=1; 2.5%) to prevent conception (Figure 1).

**Figure 2:**
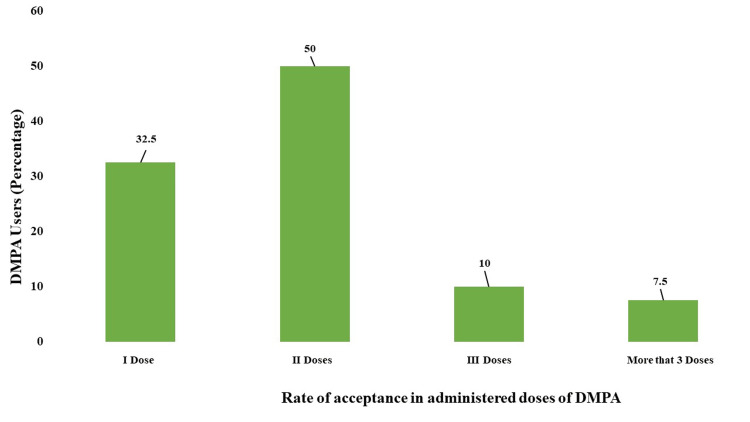
Acceptance rate of injectable contraceptive DMPA among recipients of DMPA DMPA: Depot medroxyprogesterone acetate

As shown in Figure 2, 13 participants (32.5%) opted for injectable contraceptive (DMPA) as a contraceptive method for the first time; 20 participants (50%) came for the second dose, four participants (10%) continued with the third dose, and only three participants (7.5%) continued with more than three doses.

At the initial point of data collection, it was observed that 27 (67.5%) of DMPA users adhered to subsequent doses. In this study, the primary reasons for DMPA continuation were efficacy (n=20; 50%), discreet usage (n=15; 37.5%), feeling comfortable with the contraceptive method (n=10; 25%), longer intervals between doses (n=4; 10%), easy accessibility (n=6; 15%), and curiosity about the injectable method (n=13; 32.5%). Follow-up after three months revealed that 10 (25%) of DMPA users adhered to the subsequent doses. Thus the adherence rate among these samples was found to be 20% only. Also, the study identified that half of the DMPA users reported having no side effects; 15 (37.5%) verbalized irregular bleeding, whereas 9 (22.5%) reported amenorrhea as the main reason for discontinuation of DMPA (Table 2).

**Table 2:** Factors for continuation/discontinuation of injectable contraceptive (DMPA) DMPA: Depot Medroxyprogesterone Acetate Note: This table consists of multiple responses

Continuation Factor	Frequency		%
Very effective method of contraception	20	50.0%	
Longer intervals between the doses	4	10.0%	
Comfortable with injectable contraceptive	10	25.0%	
Curiosity to adopt injectable contraceptive	13	32.5%	
Nobody knows about receiving injectable contraceptive	15	37.5%	
Easy accessibility	6	15.0%	
Experienced no side effects	20	50.0%	
Discontinuation Factor			
Physiological factor – side effects			
Amenorrhea	9	22.5%	
Irregular bleeding	15	37.5%	
Social factor			
Fertility motivation	3	7.5 %	

The study identified a significant association between the number of living children (p<0.03) and adherence to DMPA (Table 3). An association was also observed between the initiation of DMPA and occupational status (p<0.001) indicating that injectable contraceptive was used mostly by homemakers. However, age, religion, residence, and educational status, did not show any significant association with DMPA dose (p>0.05).

**Table 3:** Association between the DMPA dose (acceptance), initiation, and adherence of DMPA with demographic variables (n=40) DMPA: Depot medroxyprogesterone acetate *Significance at p<0.05 **Significance at P<0.001 Note: “0” indicates that, despite inclusion of these specific variables, there were no respondents for which they were applicable.

Baseline Variable		DMPA dose (Acceptance)					Initiation of DMPA				Adherence of DMPA		
		I Dose	II Doses	III Doses	More than III Doses	p-value	Post abortal	Postpartum	Spacing	p-value	Continuation	Discontinuation	p-value
Participant age (years)	21–25	5	3	3	3	0.185	0	2	12	0.419	5	9	0.419
	26–30	4	6	0	0		0	2	8		4	6	
	31–35	2	7	1	0		1	0	9		4	6	
	36–40	2	4	0	0		0	0	6		0	6	
	40–45	0	0	0	0		0	0	0		0	0	
Religion	Hindu	13	19	4	3	0.795	1	4	34	0.929	12	27	0.144
	Christian	0	0	0	0		0	0	0		0	0	
	Muslim	0	1	0	0		0	0	1		1	0	
Educational status of the DMPA users	High school	10	18	4	3	0.486	1	4	30	0.665	13	22	0.097
	Middle school	3	2	0	0		0	0	5		0	5	
	Primary school	0	0	0	0		0	0	0		0	0	
	Illiterate	0	0	0	0		0	0	0		0	0	
Occupational status	Shop & market sales workers	0	1	0	0	0.049 *	1	0	0	<0.001 **	1	0	0.100
	Agricultural	5	0	0	0		0	0	5		0	5	
	Home maker	8	19	4	3		0	4	30		12	22	
Number of living children in the family	1	6	2	1	0	0.204	1	1	7	0.204	3	6	0.03 *
	2	4	12	3	2		0	2	19		10	11	
	≥3	3	6	0	1		0	1	9		0	10	

## Discussion

The present study provides insights into the demographic characteristics of DMPA users which offers valuable comparisons with existing literature. Notably, a significant proportion of participants fell within the age group of 21-35 years, aligning with similar studies reporting mean ages of 26.5-27 years [18-20]. In the context of educational level, the majority of women in our study completed only higher secondary education, as seen in a study by Rajarajeswari et al. [21]. However, in other studies, such as that of Rekha et al., educational levels varied, highlighting the diverse socio-economic backgrounds of DMPA users [22]. Also, the study identified the acceptance rate for DMPA as 23.5% and an adherence rate of 67.5% at the time of initial data collection, whereas, after three months, the adherence rate further dropped to 20%. When compared with studies conducted by Rajarajeswari et al. and Rekha et al., the findings of the current study indicate differences in dropout rates. The reasons for discontinuation, irregular bleeding, and amenorrhea, align with findings from previous studies, highlighting the challenges associated with side effects and menstrual disturbances [21,22].

The current study also sheds light on motivations for DMPA usage, with the majority of participants utilizing it for spacing out pregnancies due to its efficacy and convenience. The acceptance of DMPA during the period of post-abortion and postpartum, as observed in our study, is similar to findings from Rekha et al. [22]. Furthermore, the reported reasons for continuation (i.e., efficacy and discreet usage) align with the desire for reliable and private contraceptive methods. The prevalence of irregular bleeding as a common reason for discontinuation echoes findings by Rajarajeswari et al. and Gupta et al., emphasizing the need for addressing side effects in family planning programs [19,21]. However, our study found an association between the number of living children (p<0.03) in the family with adherence to DMPA use infers its importance to prevent further pregnancies and space out their births. There was also an association between the DMPA dose and initiation of DMPA with occupational status (p<0.001). The study did not find any association with other demographic variables.

Limitations

This study primarily relied on purposive sampling, posing challenges in establishing direct causal relationships. However, it is important to note that the study's single-center design might limit the generalizability of its findings to wider populations. Being hospital-based, its conclusions may not completely represent the circumstances of the broader community.

## Conclusions

This study on DMPA acceptance, adherence, and side effects highlights its widespread acceptance, driven by factors such as efficacy and convenience, particularly in post-abortion or postpartum. Although participants reported continued usage due to the efficacy and discreet nature of DMPA, challenges with adherence were evident, with side effects (e.g., irregular bleeding and amenorrhea) significantly influencing decision-making. This result underscores the importance of improved support and counseling to address patient concerns effectively. Moreover, our findings emphasize the prevalence and impact of side effects, particularly irregular bleeding and amenorrhea, as common reasons for DMPA discontinuation, necessitating comprehensive education and support mechanisms to empower individuals in making informed choices about contraceptives. Overall, our study contributes valuable insights into DMPA usage, with implications for healthcare providers, policymakers, and individuals seeking effective contraceptive options, and advocates for enhanced support, education, and counseling to optimize reproductive health outcomes.
